# Serving the Vulnerable: The World Health Organization's Scaled Support to Countries During the First Year of the COVID-19 Pandemic

**DOI:** 10.3389/fpubh.2022.837504

**Published:** 2022-03-10

**Authors:** Micaela Pereira Bajard, Nicola Stephens, Johan Eidman, Kathleen Taylor Warren, Paul Molinaro, Constance McDonough-Thayer, Rafael Rovaletti, Shambhu P. Acharya, Peter J. Graaff, Gina Samaan

**Affiliations:** ^1^Country Strategy and Support, World Health Organization, Geneva, Switzerland; ^2^World Health Organization, Geneva, Switzerland; ^3^COVID-19 Country Technical Support, Health Emergencies Programme, World Health Organization, Geneva, Switzerland; ^4^Operational Support and Logistics, Health Emergencies Programme, World Health Organization, Geneva, Switzerland; ^5^Budget Coordination and Grant Management, Planning, Resource Coordination and Performance Monitoring, World Health Organization, Geneva, Switzerland; ^6^Health Emergencies Programme, World Health Organization, Geneva, Switzerland

**Keywords:** COVID-19, World Health Organization, country-vulnerability, United Nations, humanitarian, pandemic, emergency

## Abstract

The Inter-Agency Standing Committee (IASC), created by the United Nations (UN) General Assembly in 1991, serves as the global humanitarian coordination forum of the UN s system. The IASC brings 18 agencies together, including the World Health Organization (WHO), for humanitarian preparedness and response policies and action. Early in the COVID-19 pandemic, the IASC recognized the importance of providing intensified support to countries with conflict, humanitarian, or complex emergencies due to their weak health systems and fragile contexts. A Global Humanitarian Response Plan (GHRP) was rapidly developed in March 2020, which reflected the international support needed for 63 target countries deemed to have humanitarian vulnerability. This paper assessed whether WHO provided intensified technical, financial, and commodity inputs to GHRP countries (*n* = 63) compared to non-GHRP countries (*n* = 131) in the first year of the COVID-19 pandemic. The analysis showed that WHO supported all 194 countries regardless of humanitarian vulnerability. Health commodities were supplied to most countries globally (86%), and WHO implemented most (67%) of the $1.268 billion spent in 2020 at country level. However, proportionally more GHRP countries received health commodities and nearly four times as much was spent in GHRP countries per capita compared to non-GHRP countries ($232 vs. $60 per 1,000 capita). In countries with WHO country offices (*n* = 149), proportionally more GHRP countries received WHO support for developing national response plans and monitoring frameworks, training of technical staff, facilitating logistics, publication of situation updates, and participation in research activities prior to the characterization of the pandemic or first in-country COVID-19 case. This affirms WHO's capacity to scale country support according to its humanitarian mandate. Further work is needed to assess the impact of WHO's inputs on health outcomes during the COVID-19 pandemic, which will strengthen WHO's scaled support to countries during future health emergencies.

## Introduction

On 30 January 2020, COVID-19 was declared a public health emergency of international concern (1). The World Health Organization (WHO) launched a global COVID-19 Strategic Preparedness and Response Plan on 4 February 2020 setting out the essential pillars required to reduce transmission of the virus, save lives, and protect the vulnerable (2). COVID-19 was characterized as a pandemic on 11 March 2020. While all countries were expected to be impacted, WHO and the international community recognized that countries with pre-existing fragile settings, conflict, or humanitarian crises would be disproportionately affected. Populations in these settings have high disease comorbidity burden, crowded housing, limited access to health or socio-economic protection services, and have a low capacity to implement public health and social measures (3).

The Inter-Agency Standing Committee (IASC), created by the United Nations (UN) General Assembly in 1991, serves as the global humanitarian coordination forum of the UN system. Comprised of 18 UN and other humanitarian agencies, including WHO, the IASC recognized that some countries needed heightened levels of support to deal with the initial immediate and urgent health and non-health aspects of the pandemic, including to secure supply chains and other essential services, and to avoid disrupting ongoing operations for pre-COVID-19 humanitarian emergencies. Coordinated by the UN's Office for the Coordination of Humanitarian Affairs, the IASC initiated the Global Humanitarian Response Plan (GHRP) on 25 March 2020, covering 63 countries deemed vulnerable (4, 5). The 63 countries included those with an ongoing Humanitarian Response Plan, Refugee Response Plan, or multi-country/subregional response plan, and countries that directly requested international assistance (4).

As part of its global mandate, WHO provides support to all 194 countries to prepare for and respond to health emergencies such as pandemics. However, following the Ebola virus disease outbreak in West Africa in 2016, WHO's role in emergencies evolved so that more intense operational support could be provided to countries with fragile settings or humanitarian crises (6). World Health Organization delivers its support to countries through its global, regional, and country presence, and by working with authorities, partners, and the broader UN system (7).

Considering the scale of the COVID-19 pandemic, WHO's global health mandate including to protect the vulnerable and its extensive country presence of 149 WHO country offices at the start of the pandemic, it is important to document WHO's support to countries and to assess whether these inputs were scaled according to different contexts. This study describes WHO's inputs to countries during the first year of the pandemic and to assess whether proportionally more of the 63-target humanitarian vulnerable countries, as defined by the IASC, received WHO's support compared to other countries.

## Methods

This analysis focused on WHO's technical, commodity, and financial support to countries. We compared the proportion of GHRP (*n* = 63) and non-GHRP (*n* = 131) countries receiving WHO inputs. For actions delivered through WHO country offices, all countries with a WHO country office (*n* = 149) were included in the analysis. Nine country offices provided support to more than one country. The list of countries is provided in Supplementary Table 1.

An online survey on WHO's country office inputs and actions was sent to all WHO country offices (*n* = 149) in September 2020. Questions focused on early actions including incident management support team activation; strategic, technical, research, and logistics support to countries; as well as the WHO country office role in the UN system response. Heads of WHO country offices were asked to complete the survey covering the period 1 January to 31 August 2020. Data from each survey response were cross-checked by two WHO reviewers at headquarters and regional offices, and, where needed, data were verified by e-mail or telephone.

We compared early actions by the 149 WHO country offices by measuring actions taken either prior to the first COVID-19 case being reported in each country or before COVID-19 was characterized as a pandemic (1). Data on the first case in country were ascertained from the WHO COVID-19 Dashboard (8) which used information received through official communications under the International Health Regulations (2005) until 21 March 2020. World Health Organization dashboard data reported after this date were compiled through WHO region-specific dashboards or aggregate count data reported to WHO headquarters daily. Countries where support occurred in the same month as first case reported or same month as time of pandemic characterization were excluded from analysis due to the timing within the month being unknown.

We analyzed the data on COVID-19 health commodities made available to WHO's 194 member states globally between 1 January and 31 December 2020. These data were extracted from WHO's internal shipment report, including items requested through WHO's dedicated supply portal. Data were extracted on 29 September 2021 and validated by WHO's Operation Support and Logistics Unit. The commodities were categorized according to biomedical equipment, diagnostic kits, therapeutics, and personal protective equipment.

We also compared data on COVID-19 funds spent by WHO country offices for country level response between 1 January and 31 December 2020. The data were extracted from WHO's financial management system on 30 September 2021 (9) and validated by WHO's Planning, Resource Coordination and Performance Monitoring Department. Expenditures were categorized according to (1) coordination; (2) clinical management, diagnostics, and operational logistic support; and (3) surveillance, technical guidance, and risk communication and community engagement. Of the 149 WHO country offices, four relied on financial implementation through regional offices rather than country office. A headquarters office based in a high-income country received funds to support the country response and was therefore included in the analysis.

Data were analyzed descriptively, and proportions were compared. Analyses were conducted in STATA v17.0 (Stata Corporation, College Station, TX, USA), using chi-square tests, or Fisher's exact tests where cells were <5, to compare actions taken in GHRP countries with actions taken in non-GHRP countries. We considered differences as significant at *P* ≤ 0.05.

## Results

All 149 (100%) WHO country offices completed the survey. Table 1 shows the comparison of WHO country offices' early actions, the role of WHO country offices in the UN system response, regional or headquarter deployments to countries, and the commodity support provided, separately by country vulnerability group (GHRP/non-GHRP).

**Table 1:** WHO COVID-19 support to countries according to Global Humanitarian Response Plan country status, 2020.

	**GHRP countries n/N (%)**	**Non-GHRP countries n/N (%)**	Test of significance^*^
**EARLY ACTIONS BY WHO IN COUNTRIES**			
***Initiation of WHO Country Offices' Support to Countries Occurred Before or by the Time of the First Case Reported in Country or by the Time of Pandemic** ^**∧**^ **Characterization***			
Incident Management Support Team activated	47/63 (75)	70/86 (81)	χ^2^ = 0.99, *p* = 0.32
Conducted or supported MOH/Government in regular health sector meetings	38/46 (83)	46/68 (68)	χ^2^ = 3.17, *p* = 0.08
Supported development of the national response strategy, objectives, and operational plan	54/55 (98)	66/75 (88)	χ^2^ = 4.63, ***p*** **= 0.03**
Developed/supported response monitoring framework	32/46 (70)	34/67 (51)	χ^2^ = 3.98, ***p*** **= 0.05**
Provided expertise to MOH and partners on priority interventions related to risk communication, community engagement, disease control measures, maintaining essential health services	41/48 (85)	57/72 (79)	χ^2^ = 0.75, *p* = 0.39
Capacity-built/trained national and partner staff in technical areas	42/49 (86)	50/75 (67)	χ^2^ = 5.62, ***p*** **= 0.02**
Supported MOH or government to issue sitreps or issued sitreps or other periodic information products	30/36 (83)	37/63 (59)	χ^2^ = 6.34, ***p*** **= 0.01**
Facilitated participation in research and development activities	53/63 (84)	60/86 (70)	χ^2^ = 4.09, ***p*** **= 0.04**
Supported logistics, supply chain, and procurement	39/44 (89)	41/66 (62)	χ^2^ = 9.34, ***p*** **= 0.002**
**ROLE OF WHO IN UNITED NATIONS SYSTEM RESPONSE**			
Lead^~^ role within United Nations Country Team	54/63 (86)	71/86 (83)	χ^2^ = 0.27, *p* = 0.60
Lead^~^ coordination of UN socio-economic response	35/63 (56)	55/86 (64)	χ^2^ = 1.07, *p* = 0.30
Lead^~^ coordination of Strategic Preparedness and Response Plan	57/63 (90)	64/86 (74)	χ^2^ = 6.14, ***p*** **= 0.01**
Lead^~^ on health donor coordination	43/63 (68)	45/85 (53)	χ^2^ = 3.52, *p* = 0.06
**WHO CAPACITY IN COUNTRY**			
***WHO Workforce Surge for COVID-19***			
Technical backstopping received from **regional/sub-regional** offices	58/63 (92)	81/86 (94)	χ^2^ = 0.26, *p* = 0.61
Technical backstopping received from **headquarters**	54/63 (86)	30/86 (35)	χ^2^ = 38.20, ***p*** **< 0.001**
**CRITICAL HEALTH COMMODITIES PROCURED AND SUPPLIED TO COUNTRIES BY WHO**			
***Commodity Type***			
Biomedical equipment^**^	54/63 (86)	68/131 (52)	χ^2^ = 28.83, ***p*** **< 0.001**
Diagnostic kits	62/63 (98)	100/131 (76)	χ^2^ = 13.49, ***p*** **< 0.001**
Therapeutics^**^	18/63 (29)	5/131 (4)	χ^2^ = 22.63, ***p*** **< 0.001**
Personal protective equipment^**^	59/63 (94)	89/131 (68)	χ^2^ = 14.16, ***p*** **< 0.001**
Any of the above critical health commodities	62/63 (98)	105/131 (80)	χ^2^ = 10.36, ***p*** **< 0.001**

*GHRP, Global Humanitarian Response Plan; WHO,World Health Organization; MOH, Ministry of Health; UN, United Nations*.

* *Differences considered significant at P ≤ 0.05*.

∧ *Data was collected by month, not exact date, therefore countries where support occurred in the same month as first case reported or same month as time of pandemic characterization were excluded from analysis due to the timing within the month being unknown. This resulted in the denominator being smaller for some analyses*.

~ *Lead within United Nations Country Team (UNCT) context: chaired or co-chaired the response with United Nations Resident Coordinator; main technial agency; leading role within the UNCT*.

** *For countries in the Region of the Americas, the biomedical equipment, therapeutics and personal protective equipment data represent procurements through WHO headquarters. Further procurements were conducted by the regional office but were not available for this analysis. Bolded values are statistically significant values*.

Most WHO country offices (79%) activated their incident management support team for COVID-19 either before the individual countries reported their first cases or by the time of pandemic characterization, regardless of country vulnerability. The timing of support for priority interventions related to risk communication and community engagement, disease control measures, and maintaining essential health services was also similar across both country categories (Table 1).

A larger proportion of WHO country offices in GHRP countries supported authorities to coordinate regular health sector meetings, provided support for the development of the country's national response strategy, objectives, and operational plan; development of the country's response monitoring framework; capacity-building or training of relevant national and partner staff in technical areas such as surveillance, laboratory, and infection prevention and control; support for issuing situation reports, bulletins, or other periodic information products; and, support for logistics, supply chain, and procurement before the individual countries reported their first cases or before the time of pandemic characterization. Additionally, proportionally more WHO country offices facilitated participation in research and development activities in GHRP countries compared to non-GHRP countries (Table 1).

Most WHO country offices in GHRP and non-GHRP countries played a lead role in the UN Country Team for the COVID-19 response and in the coordination of the “Health First” pillar of the UN framework for the immediate socio-economic response for COVID-19 (10). Proportionally more WHO country offices in GHRP countries played a lead role in the coordination of health donors and coordination of the strategic preparedness and response plan (Table 1).

Most countries, regardless of vulnerability, received deployments from regional offices. Proportionally more GHRP countries received deployment surge support from headquarters compared to non-GHRP countries (Table 1).

World Health Organization procured and supplied critical COVID-19 health commodities to 167/194 (86%) countries globally, with a significantly higher proportion of GHRP countries receiving commodities when compared to non-GHRP countries (Table 1).

**Figure 1:**
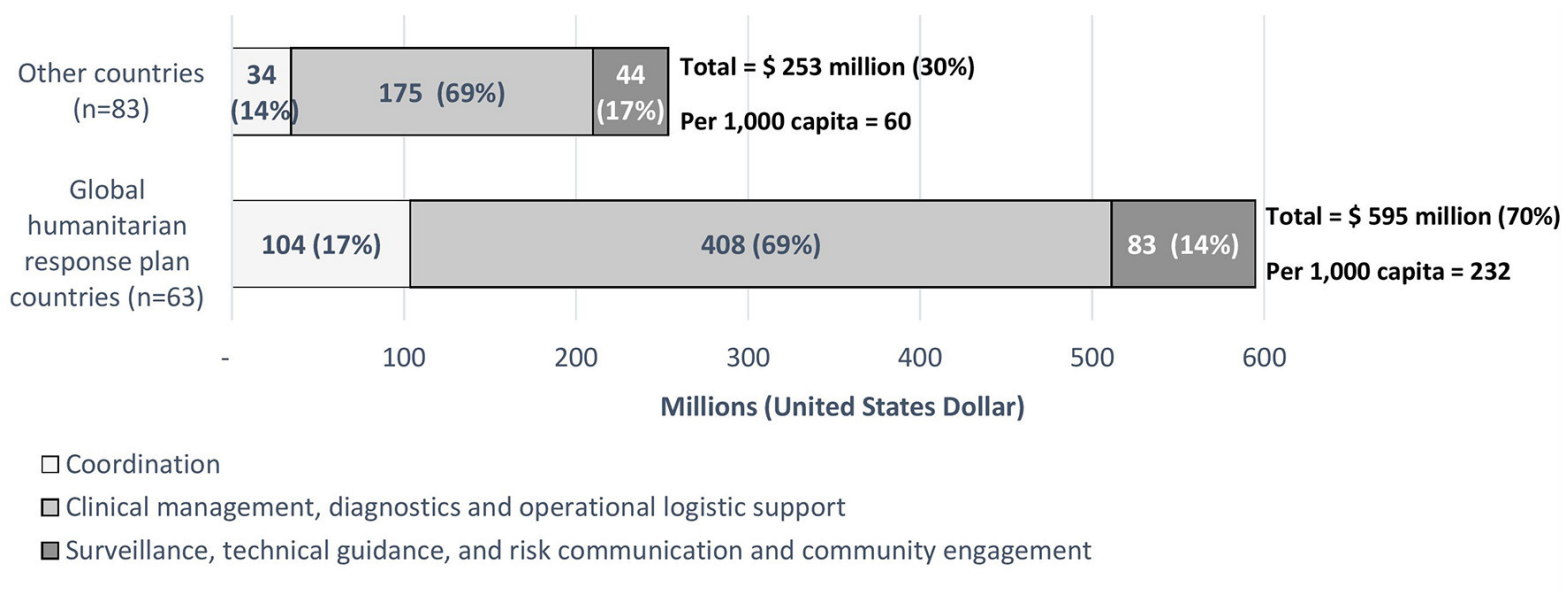
WHO's country office financial support to countries for COVID-19 in 2020 comparing the Global Humanitarian Response Plan target countries to other countries.

In 2020, WHO utilized US $ 1.268 billion for the COVID-19 response, of which US $ 848 million (67%) were implemented through 146 WHO country offices to support country level preparedness and response actions (Figure 1). Over twice the volume of funds were utilized in GHRP countries compared to non-GHRP countries (70 vs. 30%) (Figure 1). Per 1,000 capita, nearly four times as much was spent in GHRP compared to non-GHRP countries (Figure 1). The type of financial implementation was similar for both country groups, with most funds utilized to support clinical management including infection prevention and control, diagnostics, and commodities. However, the magnitude of that investment was greater for GHRP countries.

## Discussion

Two key themes emerge from this analysis. First, WHO supported countries regardless of country vulnerability status, and second, WHO did provide operational support to proportionally more countries with humanitarian vulnerability as defined by the IASC. With the mandate to promote health, keep the world safe, and to serve the vulnerable (11), WHO's strategy during the pandemic was to mobilize the entire Organization—headquarters, regional offices, and country offices alike—in support of a country-focused response. This strategic intent was demonstrated by the magnitude of financial support implemented at country level, and deployments to over 90% of countries with a WHO country office.

World Health Organization serves a unique normative, technical, and convening role globally (12), which is critical to prepare for and respond to an emerging disease. All countries benefited from technical guidance on disease control, risk communication, and community engagement, and maintenance of essential health services. Through WHO country offices, countries received technical and financial support for preparedness and response actions, essential health commodities, and leadership to mobilize through the UN system. COVID-19 highlighted that vulnerability can be universal, affecting countries of all income levels. As noted by the Independent Panel for Pandemic Preparedness and Response, WHO country offices should be sufficiently resourced and equipped to respond to the pandemic preparedness and response requests from national authorities (12). Strengthening and enabling WHO country offices will improve the resilience of countries for future emergencies.

The COVID-19 pandemic exacerbated inequalities that increase the risk of conflict and humanitarian suffering (13) and has overwhelmed health systems (14). Weak health systems and protracted conflict result in many countries being unable to deliver basic health, nutrition, and social services, and there are significant gaps in the capacity of many vulnerable countries to manage health emergencies (15). World Health Organization's scaled support to countries was clearly demonstrated across many country-level technical actions, in the coordination of strategic preparedness and response plans, and in the provision of essential health commodities. Although WHO financially supported all countries, the scale of support was much greater in countries with humanitarian vulnerability. Compared to offices in non-GHRP countries, a significantly larger proportion of WHO country offices in GHRP countries provided early leadership support, suggesting the importance of strong country-level partnerships in vulnerable countries and capitalizing on existing, well-established coordination, and response mechanisms (16) for public health and humanitarian crises.

This study showed that WHO country offices supported both vulnerable and other countries in research and development activities, indicating WHO's commitment to advancing equity in health research. The after-action review (17) of the 2009 influenza pandemic recommended that research protocols be developed to facilitate all countries to rapidly gather and contribute knowledge in pandemic preparedness and response. Several WHO initiatives including the “R&D Blueprint for Action to Prevent Epidemics” (18) and the “Pandemic Influenza Special Investigations and Studies” (19) have since been established by WHO to advance this agenda and strengthen research capacities among low- and middle-income countries. During COVID-19, research protocols such as the Unity Studies (20) and Solidarity Trials (21) were developed, and operational support was provided to countries for implementation.

Our analysis focused on vulnerability of countries from the perspective of a humanitarian mandate. The list of 63 countries deemed vulnerable was rapidly consolidated by the IASC soon after COVID-19 was characterized as a pandemic. Our results show the benefit of rapidly defining vulnerable contexts at the outset of the pandemic, as it translated to a greater scale of technical, commodity, and financial support channeled to them. Recognizing that vulnerability can be assessed in many ways, including through equity-focused qualitative and quantitative measures, a key observation is to conduct rapid vulnerability assessments so that global action and solidarity can be mobilized quickly to address the needs of the vulnerable.

This study has limitations. First, the focus is on WHO's inputs based on self-report and logistics and financial data. The study did not capture the perspective of countries and partners or the public health impact of WHO's inputs and actions. Understanding the trajectory, the evolving role of WHO country offices over the course of the pandemic, and the outcome of support, is critical to drive improvement and to enable WHO country offices to do better in future emergencies. While this has been explored for a subset of countries qualitatively (22, 23), further data and research are needed to enrichen the understanding of WHO's operational preparedness and response impact. Second, nine country offices provided support to more than one country, therefore the results of the analyses may not fully reflect the direct support to each country served by these offices. Third, this study explored WHO's inputs at country level using the IASC's pre-defined list of countries deemed to have humanitarian vulnerability. Data were not available to assess population level vulnerabilities or outcomes. Therefore, conclusions cannot be drawn about the impact of investments or whether vulnerable populations in different countries were served by WHO's inputs proportional to their needs.

The COVID-19 pandemic has impacted health, economies, communities, and individuals in a way no other public health emergency has before, with a particularly severe impact on vulnerable countries and populations (6, 13, 14). The pandemic's collateral damage in 2020 includes an increase in tuberculosis deaths for the first time in over a decade (24) and an estimated 60% increase in reports of gender-based violence coinciding with movement restrictions (25). Disparities continued in 2021 with inequities in vaccine deployment leaving high-risk groups in many countries unprotected (26). World Health Organization continues to support all countries globally and provide intensified support to countries with fragile settings, conflict, or humanitarian crises. Global leadership, solidarity including sustainable financing and commitment to its humanitarian mandate are critical for when future emergencies arise.

## Data Availability Statement

The list of countries is provided as Supplementary Table 1. Survey data are not publicly available due to country identifiability. Further inquiries can be directed to the corresponding author/s.

## Author Contributions

GS, MPB, JE, KW, SA, and PG: conceptualization. MPB, PM, CM-T, RR, GS, JE, and NS: data collection, analysis, and interpretation. NS, GS, and MPB: drafting the manuscript. GS, MPB, NS, JE, KW, PM, CM-T, RR, SA, and PG: review and revision of manuscript. All authors contributed to the article and approved the submitted version.

## Conflict of Interest

The authors declare that the research was conducted in the absence of any commercial or financial relationships that could be construed as a potential conflict of interest.

## Publisher's Note

All claims expressed in this article are solely those of the authors and do not necessarily represent those of their affiliated organizations, or those of the publisher, the editors and the reviewers. Any product that may be evaluated in this article, or claim that may be made by its manufacturer, is not guaranteed or endorsed by the publisher.
